# Case report: Three contrasting presentations of acute oesophageal necrosis and the determinants of outcome

**DOI:** 10.3389/fmed.2026.1882540

**Published:** 2026-07-15

**Authors:** Guo Hou Loo, Nik Aisyah Kosai, Val Michael Villareal, Guhan Muthukumaran, Nik Ritza Nik Mahmood

**Affiliations:** 1 Upper Gastrointestinal, Metabolic and Bariatric Surgery Unit, Department of Surgery, Faculty of Medicine, Hospital Canselor Tuanku Muhriz, Universiti Kebangsaan Malaysia, Kuala Lumpur, Malaysia; 2 Department of Surgery, Royal Surrey County Hospital NHS Foundation Trust, Guildford, United Kingdom

**Keywords:** acute oesophageal necrosis, black oesophagus, case report, critical illness, endoscopic hemostasis, Gurvits syndrome, upper gastrointestinal bleeding

## Abstract

**Background:**

Acute oesophageal necrosis (AEN), or “black oesophagus,” is a rare but potentially lethal cause of upper gastrointestinal bleeding (UGIB), classically described in elderly men with cardiovascular comorbidity. Contemporary reports emphasize a broader clinical spectrum encompassing younger immunosuppressed and critically ill patients.

**Case description:**

We describe three patients managed at a single tertiary Malaysian center. Case 1: a 77-year-old man with type 2 diabetes and stage 3b chronic kidney disease, not on anticoagulation, presented with coffee-ground vomitus; endoscopy revealed circumferential blackish necrosis of the distal two-thirds of the oesophagus; he was treated with submucosal adrenaline injection and supportive care and achieved complete mucosal healing at 6 weeks. Case 2: a 52-year-old woman with relapsed focal segmental glomerulosclerosis on prednisolone and ciclosporin, complicated by methicillin-sensitive *Staphylococcus aureus* bacteraemia, hospital-acquired pneumonia, and respiratory failure requiring intubation. On the eighth day of admission she developed haematemesis with blood-stained oropharyngeal secretions, prompting emergency bedside oesophagogastroduodenoscopy; this demonstrated severe AEN with extensive submucosal tear, intramural haematoma, and multifocal necrosis, managed conservatively with nasojejunal feeding and high-dose proton pump inhibitor, with full mucosal recovery by 76 days. Case 3: a 49-year-old woman with end-stage renal failure on continuous ambulatory peritoneal dialysis (CAPD) for 6 years, admitted with *relapsing Acinetobacter baumannii* peritonitis, complicated by hospital-acquired carbapenemase-producing *Klebsiella pneumoniae* peritonitis. Her Tenckhoff catheter was removed early and she was converted to haemodialysis; she met Sepsis-3 criteria and required intensive care. On day 49 she developed UGIB; eight sequential endoscopies demonstrated a large oesophageal clot, extensive sloughy necrotic mucosa with longitudinal ulceration, and recurrent haemorrhage requiring multimodal endoscopic hemostasis (endoclips, adrenaline, hemoblock, hemospray, tranexamic acid), with ultimate mucosal healing. Despite this, she died on day 91 from septic shock attributed to a refractory intra-abdominal infection and catheter-related candidaemia; an indirect contribution from her complicated oesophageal course cannot be excluded.

**Conclusion:**

These three contrasting cases illustrate the heterogeneity of AEN and reinforce the contemporary teaching that long-term outcome is determined principally by the underlying systemic illness. AEN should be considered in any acutely unwell patient with UGIB regardless of demographic profile, including immunosuppressed and long-term dialysis recipients.

## 1 Introduction

Acute oesophageal necrosis (AEN), also known as “black oesophagus,” acute necrotizing oesophagitis, or Gurvits syndrome, is a rare disorder first described by Goldenberg et al. in 1990 (1) and subsequently characterized as a distinct clinical entity, with an endoscopic staging system, by Gurvits et al. in 2007 (2). It is defined endoscopically by circumferential, diffuse, blackish discoloration of the oesophageal mucosa, most pronounced in the distal third and terminating abruptly at the gastro-oesophageal junction (3). The reported prevalence ranges from 0.01 to 0.28% of upper endoscopies (4, 5), with peak incidence in the seventh decade and a male-to-female ratio of approximately four to one (6).

The pathogenesis is widely conceptualized as a “two-hit” process: a low-flow ischemic insult to the watershed distal oesophagus, superimposed on topical caustic injury from massive reflux of gastric contents in a host with impaired mucosal defense (3, 7, 8). Predisposing conditions include advanced age, diabetes, atherosclerotic cardiovascular disease, chronic kidney disease, malignancy, hemodynamic instability, sepsis, malnutrition, alcohol misuse, and gastric outlet obstruction (3, 6, 9). Recent reports have expanded the spectrum to include post-cardiac-arrest hypoperfusion (10), the post-operative state and chemotherapy (11), and immunosuppression after solid-organ transplantation (12, 13). AEN in patients on long-term peritoneal dialysis has been only rarely documented (14).

Historical mortality of 30–32% largely reflects the burden of underlying comorbidity; disease-specific mortality is below 6% (6, 9, 15). We present three contrasting cases of AEN managed at a single tertiary referral center in Malaysia to illustrate the heterogeneity of presentation, the breadth of endoscopic intervention required, and the contemporary teaching that long-term outcome is determined by the underlying systemic illness rather than by the oesophageal injury itself. This report follows the CARE guidelines for case reports.

## 2 Case descriptions

### 2.1 Case 1: classical AEN in an elderly comorbid man

A 77-year-old man with longstanding type 2 diabetes mellitus and stage 3b chronic kidney disease (estimated glomerular filtration rate 35 mL/min/1.73 m^2^) presented to the emergency department with 2 days of coffee-ground vomitus and epigastric discomfort. He was not on therapeutic anticoagulation, antiplatelet therapy, or non-steroidal anti-inflammatory drugs. He denied frank hematemesis, dysphagia, odynophagia, or recent ingestion of caustic agents; he was a non-smoker and lifelong teetotaller. On examination, he was hemodynamically stable (blood pressure 132/76 mmHg, heart rate 84 beats per minute) with mild epigastric tenderness. Investigations demonstrated normocytic anemia (hemoglobin 6.7 g/dL), random blood glucose of 15 mmol/L, and no metabolic acidosis. He was resuscitated with crystalloids and two units of packed red cells (post-transfusion hemoglobin 9.4 g/dL), started on intravenous esomeprazole (80 mg bolus then 8 mg/h infusion), and kept nil per oral.

Oesophagogastroduodenoscopy (OGDS) within 24 h revealed diffuse, circumferential blackish discoloration of the oesophageal mucosa from the gastro-oesophageal junction to the middle third, with a sharp transition at the gastro-oesophageal junction (Figure 1A). A longitudinal mucosal break with exposed underlying muscle and active oozing at the distal oesophagus was treated with submucosal adrenaline (1:10,000) injection, achieving durable hemostasis. Biopsy was deferred owing to mucosal friability. Conservative management with bowel rest for 72 h, intravenous esomeprazole for 72 h followed by oral esomeprazole 40 mg twice daily for 8 weeks, and oral sucralfate 1 g four times daily for 4 weeks was continued. He was transitioned to a soft diet from day 4 and discharged on day 8. Surveillance OGDS at 6 weeks demonstrated complete mucosal healing (Figure 1B), and he remained asymptomatic at three-month review.

**Figure 1A:**
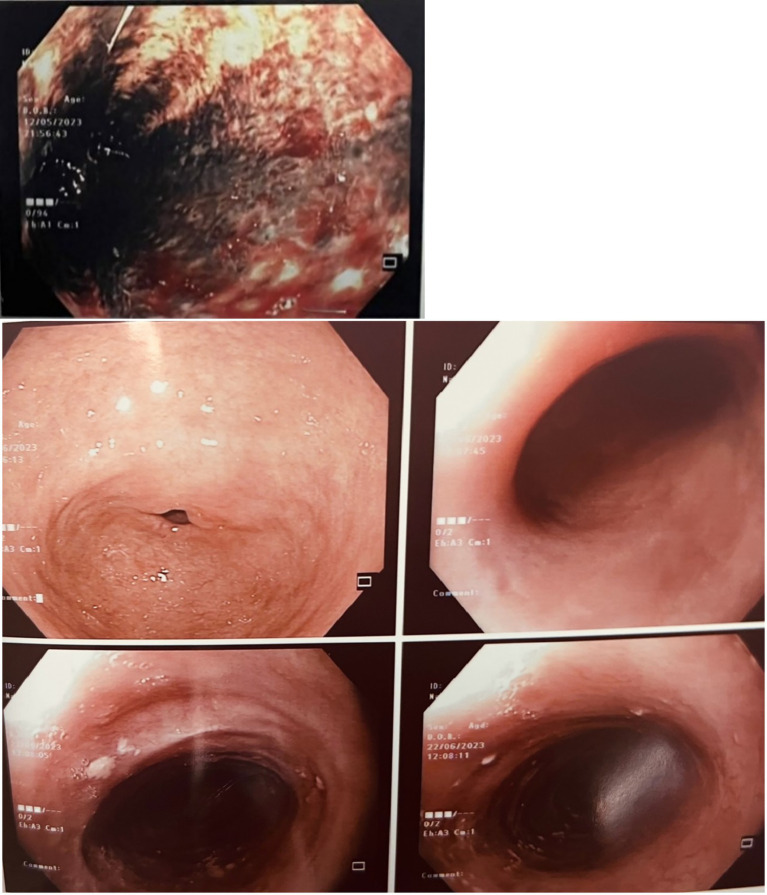
Case 1 - Index OGDS showing diffuse, circumferential blackish discolouration of the oesophageal mucosa consistent with acute oesophageal necrosis. A longitudinal mucosal break with exposed underlying muscle and active oozing is visible in the distal oesophagus.

**Figure 1B:**
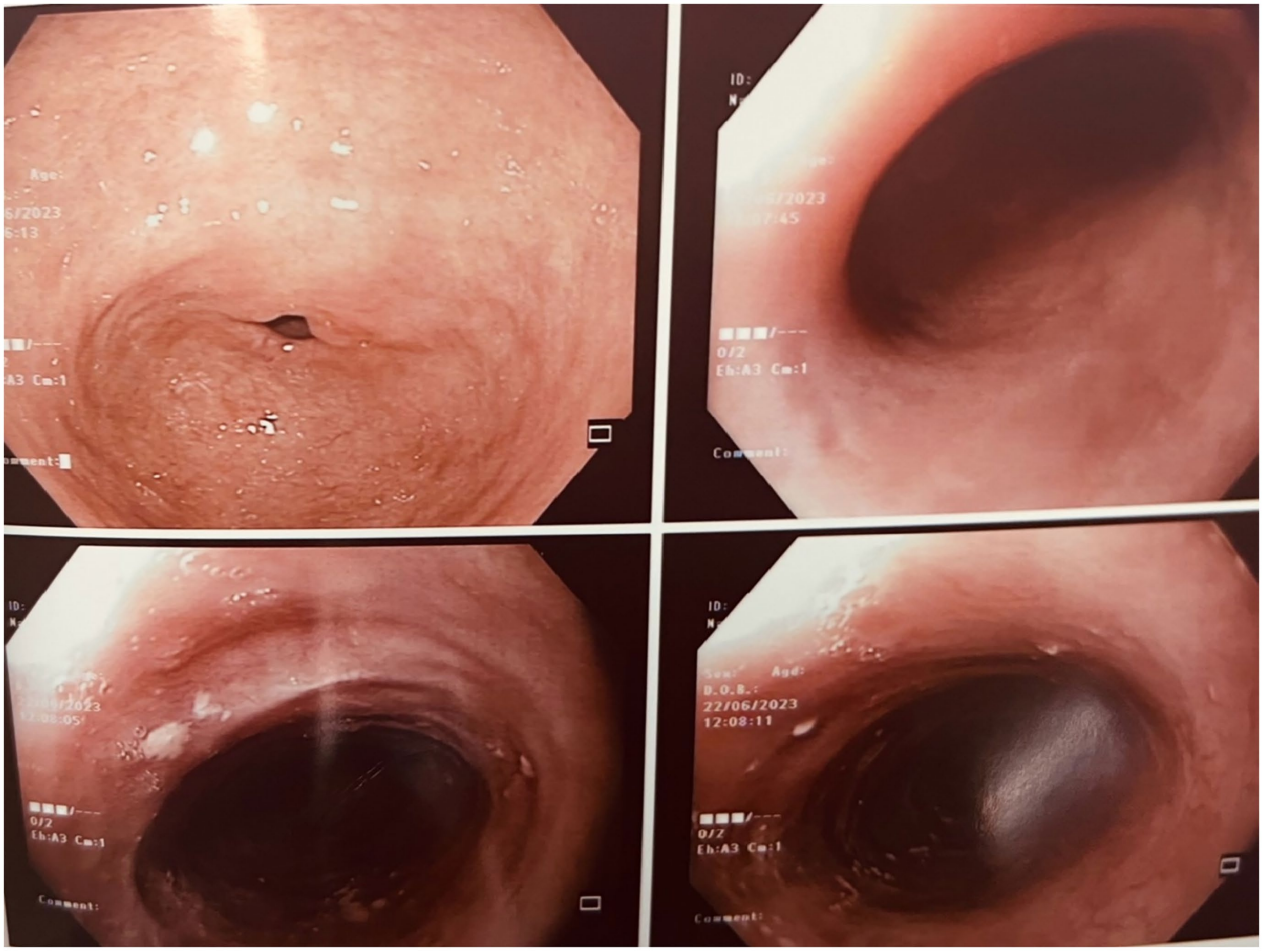
Case 1–surveillance OGDS at 6 weeks demonstrating complete mucosal healing with restoration of the normal vascular pattern. (a) Healed gastro-oesophageal junction; (b–d) progressive views of the lower, middle, and upper oesophagus, without residual ulceration, stricture, or stenosis.

### 2.2 Case 2: severe AEN in a younger immunosuppressed woman with sepsis-driven critical illness

A 52-year-old Malay woman was admitted with a relapse of biopsy-proven focal segmental glomerulosclerosis (FSGS), first diagnosed 3 years prior to the index admission and treated with intravenous cyclophosphamide followed by maintenance ciclosporin and prednisolone, with one previous nephrotic relapse 2 years prior to admission and a recent herpes zoster infection that had necessitated transient withdrawal of ciclosporin. At admission, she was on prednisolone 45 mg once daily and ciclosporin 50 mg twice daily following recent escalation of her steroid dose for persistent proteinuria, oedema, and a rising serum creatinine. Her regular medications were prednisolone, ciclosporin, and antihypertensives; she was not on therapeutic anticoagulation or antiplatelet therapy. She presented with progressive abdominal distension, lower-limb oedema, and respiratory discomfort. Initial investigations revealed profound hypoalbuminaemia (17 g/L), acute-on-chronic kidney injury (creatinine 208.9 μmol/L from baseline 151.6 μmol/L), and significant proteinuria. During admission, she developed lower-limb cellulitis with methicillin-sensitive *Staphylococcus aureus* (MSSA) bacteraemia treated with cefazolin, and subsequently hospital-acquired pneumonia with type 1 respiratory failure necessitating intubation and intensive care; intravenous meropenem was added.

**Figure 2:**
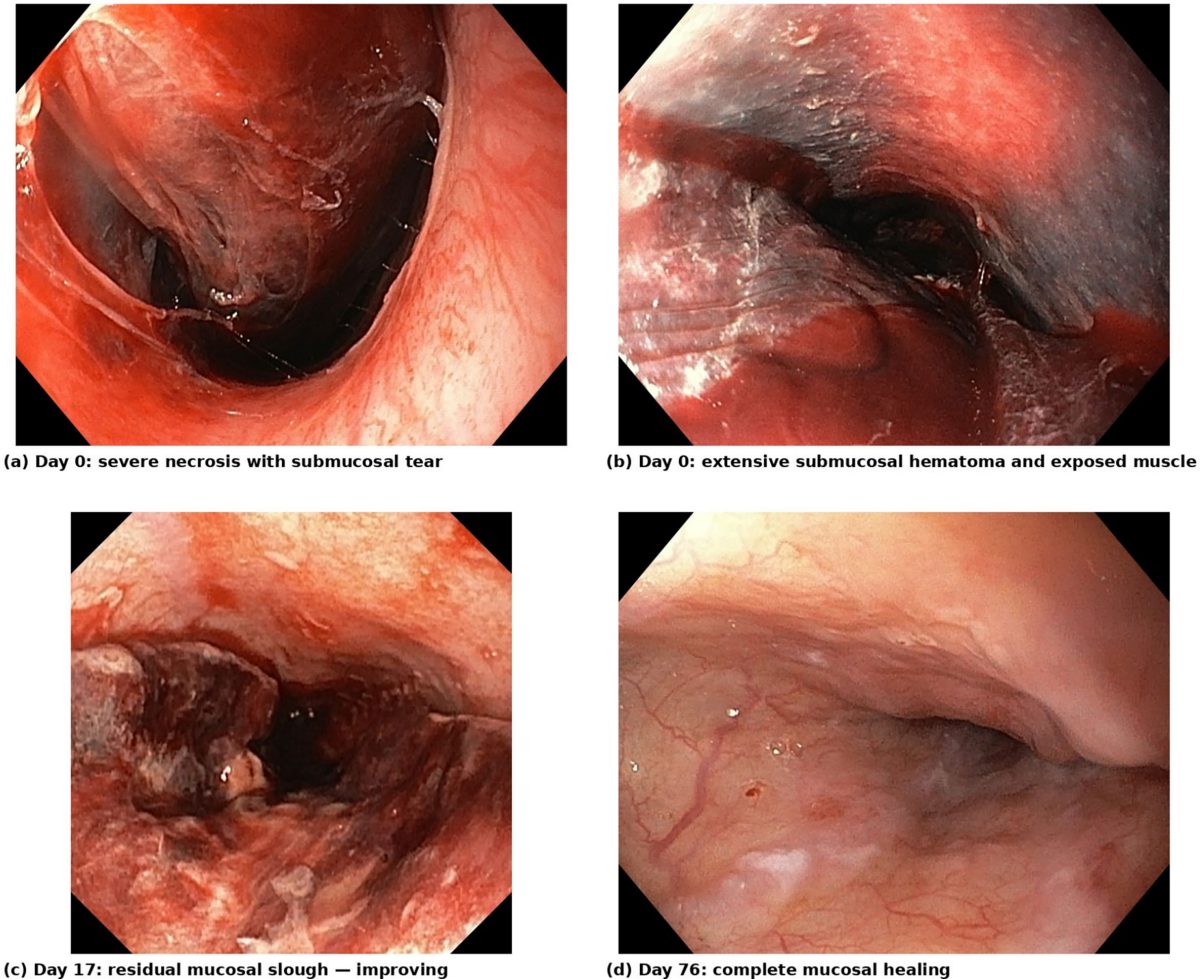
Case 2–sequential OGDS showing temporal evolution. **(a)** Day 0: severe circumferential necrosis with a deep submucosal tear at the distal oesophagus and active bleeding. **(b)** Day 0: extensive submucosal haematoma with exposed muscle visible as the silvery–grey base of the defect. **(c)** Day 17: residual mucosal slough in the upper and middle thirds with marked overall improvement and a normal-appearing lower third. **(d)** Day 76: complete mucosal healing with restoration of the normal vascular pattern and no evidence of stricture or stenosis.

On the eighth day of admission, she developed haematemesis with blood-stained oropharyngeal secretions in the context of progressive oliguria, oedema, and ongoing sepsis despite vasopressor support, having received five units of packed red cells in the preceding 24 h. Emergency bedside OGDS demonstrated massive intraluminal blood clots from the upper oesophagus to the gastro-oesophageal junction, an extensive area of unhealthy black mucosa from the gastro-oesophageal junction with extensive submucosal tear and intramural haematoma, and multiple discrete patches of mucosal necrosis (Figures 2a,b). No endoscopic intervention was attempted on the diffusely friable necrotic mucosa; a nasojejunal tube was placed endoscopically under direct vision and anchored at 90 cm. Because the diffusely friable necrotic mucosa precluded safe endoscopic hemostasis, contrast-enhanced computed tomography (CT) angiography of the thorax was performed immediately after the index endoscopy to identify any arterial source of active intra-thoracic bleeding amenable to interventional-radiological embolization, to exclude oesophageal perforation, and to detect any mediastinal collection. The CT demonstrated a distended oesophagus with an intraluminal clot but no contrast extravasation, no perforation, and no mediastinal collection, supporting a conservative non-operative approach. She was managed conservatively with continuous intravenous esomeprazole infusion (8 mg/h for 48 h, then 40 mg twice daily, continued for 12 weeks), source control of sepsis, and judicious post-pyloric jejunal feeding. Surveillance OGDS at 17 days demonstrated upper- and middle-third mucosal slough with marked improvement (Figure 2c), and at 76 days the oesophageal mucosa was fully restored (Figure 2d). She subsequently recovered from the systemic insult and remained asymptomatic at outpatient follow-up.

### 2.3 Case 3: severe recurrent-bleeding AEN in a long-term peritoneal-dialysis patient

A 49-year-old Malay woman, a primary-school mathematics teacher, was admitted on day 1 (28 November 2025) with relapsing *Acinetobacter baumannii* peritoneal-dialysis peritonitis. She had been on continuous ambulatory peritoneal dialysis (CAPD) for 6 years for end-stage renal failure secondary to a missed glomerulonephritis, and was not on therapeutic anticoagulation or antiplatelet therapy. She had had a recent admission for *A. baumannii* peritonitis with septic shock, and the present relapse again presented with septic shock requiring noradrenaline. Because of relapsing, refractory peritonitis, the Tenckhoff catheter was removed on day 5 (2 December 2025) in accordance with ISPD criteria, and she was converted to intermittent haemodialysis from day 8 (5 December 2025) via a femoral catheter; thus, by the time of the upper gastrointestinal bleed described below she was no longer receiving peritoneal dialysis. Her prolonged admission was subsequently complicated by hospital-acquired carbapenemase-producing *Klebsiella pneumoniae* peritonitis with an intra-abdominal collection, and later by *Enterococcus* peritonitis. The full chronology is summarized in Table 1.

**Table 1:** Chronology of Case 3, expressed as days from the index admission (28 November 2025) with calendar dates in parentheses. CP-CRE, carbapenemase-producing Enterobacteriaceae; ISPD, International Society for Peritoneal Dialysis; MBL, metallo-β-lactamase; OGDS, oesophagogastroduodenoscopy; PD, peritoneal dialysis; RICU, renal intensive care unit; UGIB, upper gastrointestinal bleeding. Note that the Tenckhoff catheter was removed and the patient converted to haemodialysis (days 5–8) well before the onset of the oesophageal injury (day 49).

Day (from index admission)	Calendar date	Key events
Day 1	28 November 2025	Index admission–relapsing *A. baumannii* PD peritonitis with septic shock (noradrenaline); RICU; intraperitoneal ceftazidime + cloxacillin
Day 5	2 December 2025	Tenckhoff catheter removed for refractory peritonitis (ISPD criteria); IV ampicillin–sulbactam
Day 8	5 December 2025	Femoral catheter inserted; converted to intermittent haemodialysis
Day 20	17 December 2025	OGDS for anemia workup–gastritis only (oesophagus not necrotic at this point)
Day 29	26 December 2025	Hospital-acquired CP-CRE (MBL) *K. pneumoniae* peritonitis + intra-abdominal collection; percutaneous drainage; IV meropenem
Day 34	31 December 2025	Polymyxin B salvage added
Day 39	5 January 2026	Tigecycline added (combination salvage)
Day 43	9 January 2026	Ceftazidime–avibactam + aztreonam started (26-day course; for MBL-producing strain)
Day 49	15 January 2026	First UGIB; OGDS 1–large 15-cm adherent clot, longitudinal ulceration; high-dose IV esomeprazole
Day 53	19 January 2026	OGDS 2–clot partially cleared; ulceration, erosion, and necrotic mucosa 28–33 cm (part of the AEN); nasogastric tube repositioned under direct vision; no active bleeding
Day 54	20 January 2026	OGDS 3–fresh bleeding 7 o’clock; 4 endoclips + submucosal adrenaline + Hemoblock® (interval re-bleed)
Day 57	23 January 2026	OGDS 4–sloughy necrotic mucosa 22–28 cm; adrenaline + Hemoblock® + Hemospray®; IV tranexamic acid; coagulopathy corrected
Day 60	26 January 2026	OGDS 5–bleeding site inactive; sustained hemostasis; nasogastric tube placed
Day 71	6 February 2026	OGDS 6–recurrent mid-oesophageal bleed; Hemoblock®; transient suspected cavitation (negative under-water test) (interval re-bleed); intubated for hospital-acquired pneumonia
Day 74	9 February 2026	Peritonitis evolves to Enterococcus species
Day 75	10 February 2026	Catheter-related candidaemia (central-line yeast; peripheral culture negative)
Day 76	11 February 2026	OGDS 7–nasojejunal feeding tube sited alongside retained clip
Day 78	13 February 2026	Central venous catheter removed; IV anidulafungin started (later de-escalated to fluconazole)
Day 81	16 February 2026	OGDS 8–mucosal healing (Gurvits Stage 3); new nasojejunal tube via enteroscopy; dialysis withdrawn after family discussion
Day 91	26 February 2026	Death–septic shock (refractory intra-abdominal infection + candidaemia); oesophageal mucosa healing

#### 2.3.1 Severity assessment

At the index admission (day 1), the patient was in septic shock requiring noradrenaline and met Sepsis-3 criteria, and she was managed in the renal intensive care unit (16). Over the prolonged admission she required repeated periods of vasopressor support (noradrenaline up to 0.2 μg/kg/min) during successive septic episodes, and developed multiple organ dysfunction including acute transaminitis, coagulopathy requiring fresh-frozen plasma, and, later, type 1 respiratory failure necessitating intubation. Inflammatory markers were markedly deranged during the septic episodes (peak procalcitonin >100 ng/mL; C-reactive protein up to approximately 15 mg/dL). Formal sequential SOFA scores and serum lactate were not systematically recorded in the medical record.

#### 2.3.2 Microbiological and source-control management

The index relapsing *A. baumannii* peritonitis was treated initially with intraperitoneal ceftazidime and cloxacillin, then intravenous ampicillin–sulbactam (escalated to 6 g twice daily to cover multidrug-resistant organisms) for 14 days after Tenckhoff-catheter removal. On day 29 (26 December 2025), peritoneal-fluid culture grew a carbapenemase-producing *K. pneumoniae* (subsequently characterized as a metallo-*β*-lactamase–producing strain, resistant to ceftazidime–avibactam and only intermediate to colistin), in association with an intra-abdominal collection requiring serial percutaneous drainage. In accordance with the 2022 ISPD peritonitis-management recommendations (17), this was treated with intravenous meropenem (6 days), followed by combination salvage therapy with polymyxin B and tigecycline, and subsequently a 26-day course of ceftazidime–avibactam plus aztreonam (the recommended combination for metallo-*β*-lactamase–producing Enterobacterales). Source control of the intra-abdominal collection was never fully achieved despite multiple percutaneous drains. Surrogate evidence against active oesophageal infectious superinfection was sought during the bleeding episodes: serial routine and fungal blood cultures, central-catheter-tip cultures, peritoneal-fluid cultures, and cross-sectional imaging of the chest and abdomen; mucosal biopsy was deferred throughout because of mucosal friability and the prohibitive perforation risk.

**Figure 3:**
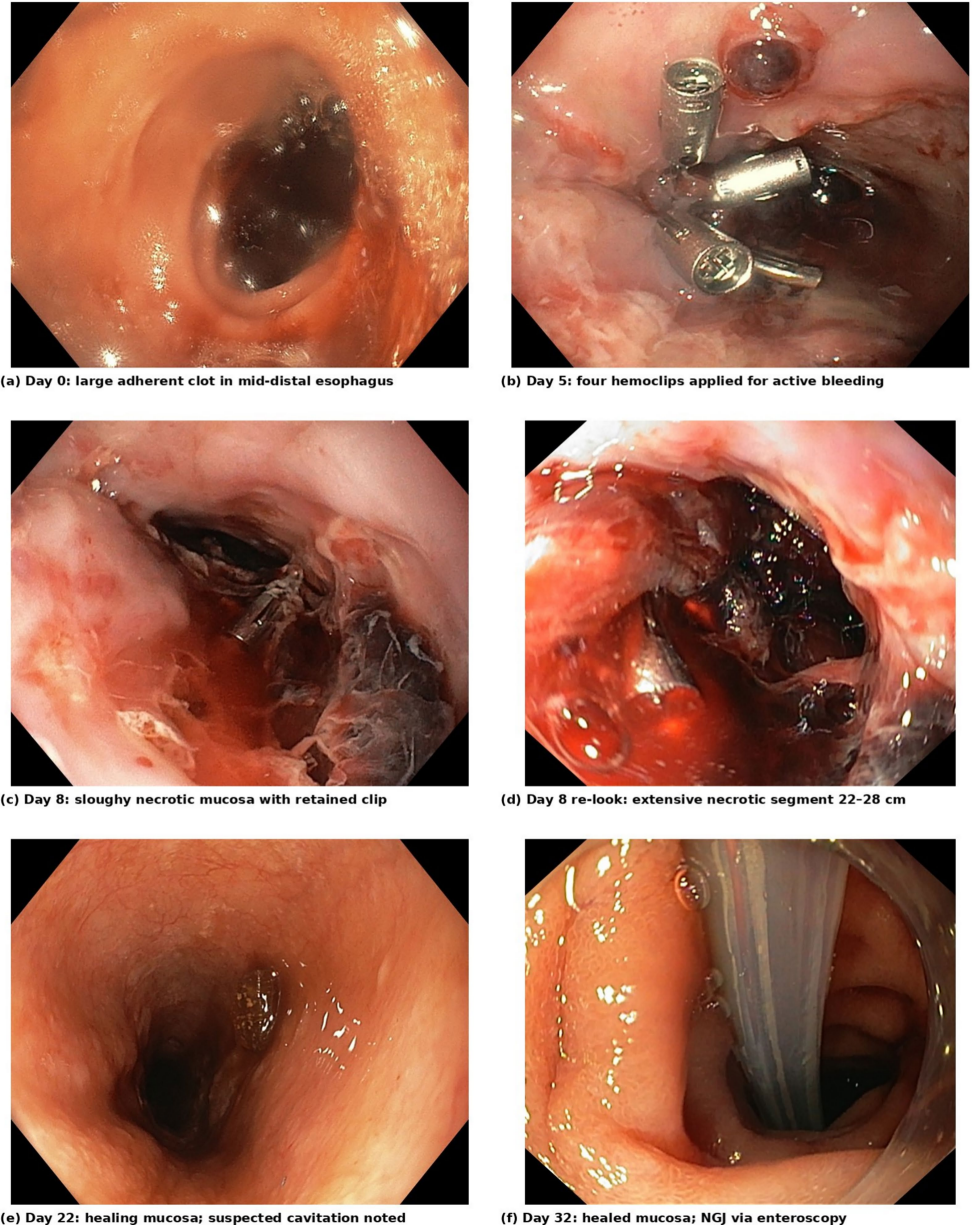
Case 3–sequential OGDS demonstrating recurrent UGIB, multimodal endoscopic hemostasis, and eventual mucosal healing in the context of dialysis-associated peritonitis and prolonged critical illness. Days are expressed from the index admission (see Table 1). **(a)** Day 49 (15 January): large adherent clot occupying the mid- to distal oesophagus, with extensive longitudinal ulceration. **(b)** Day 54 (20 January): four endoclips deployed for active bleeding adjacent to the haematoma, supplemented with submucosal adrenaline and Hemoblock® spray. **(c)** Day 57 (23 January): sloughy necrotic mucosa with retained endoclip. **(d)** Day 57 re-look (23 January) by the upper gastrointestinal surgical team: extensive sloughy necrotic segment 22–28 cm from the incisor with active oozing. **(e)** Day 71 (6 February): substantial healing with the residual clip *in situ*; a small cavitation distal to the clip raised the possibility of a fistula tract, but under-water submersion demonstrated no air bubbling, arguing against a free communicating fistula. **(f)** Day 81 (16 February): mucosal healing; a new nasojejunal feeding tube has been placed via enteroscopy for the management of a blocked NGJ tube.

On day 49 (15 January 2026), she developed coffee-ground aspirate and hematemesis. The first of eight sequential endoscopic procedures performed over the subsequent 32 days demonstrated a large adherent clot (approximately 15 cm) occupying ≥70% of the oesophageal lumen from the mid- to distal oesophagus, with the visible distal segment showing extensive longitudinal ulceration; large clots also occupied the gastric fundus and second part of the duodenum, and the index clot could not be flushed out (Figure 3a). Esomeprazole was initiated as an 80 mg intravenous bolus followed by continuous infusion at 8 mg/h for 72 h, then transitioned to 40 mg intravenous twice daily; the continuous infusion was reinstituted for a further 72 h during each subsequent re-bleeding episode. Coagulopathy emerging during her septic course was corrected with fresh-frozen plasma and platelet support to target an international normalized ratio (INR) < 1.5 and a platelet count >80 × 10^9^/L before each endoscopic intervention.

She developed recurrent bleeding and underwent seven further endoscopic procedures (Table 1). On day 53 (19 January), the clot at 28 cm was partially suctioned and cold-snared, revealing ulceration, erosion, and necrotic mucosa with hematoma extending from 28 to 33 cm, consistent with the evolving acute oesophageal necrosis, with no active bleeding from that segment; the nasogastric tube was repositioned under direct endoscopic vision and re-anchored. On day 54 (20 January), fresh bleeding at the 7 o’clock position proximal to the hematoma was treated with four endoclips, 8 mL of submucosal adrenaline, and Hemoblock® spray (Figure 3b). On day 57 (23 January), a large clot over the distal oesophagus with sloughy necrotic mucosa from 22 to 28 cm and slow oozing was treated with submucosal adrenaline, Hemoblock®, and Hemospray®; intravenous tranexamic acid was added and coagulopathy corrected (Figures 3c,d). On day 60 (26 January), the previously bleeding site was confirmed inactive and a nasogastric tube was placed under direct vision, marking sustained hemostasis. On day 71 (6 February), a recurrent bleed from a raw mucosal ulcer at the 3 o’clock position in the mid-oesophagus was treated with Hemoblock®; a small distal cavitation at the 6 o’clock position raised concern for a possible fistula tract, although under-water submersion demonstrated no air bubbling, arguing against a free communicating fistula (Figure 3e). On day 76 (11 February), a nasojejunal feeding tube was sited alongside the retained clip. The final OGDS on day 81 (16 February) demonstrated that the previous oesophageal ulceration was healing, and a new nasojejunal tube was placed via enteroscopy (Figure 3f). The oesophageal injury thus progressed through Gurvits Stage 1 (hemorrhagic necrosis) at presentation, to Stage 2 (sloughy mucosa) over days 57–71, to Stage 3 (mucosal restoration) by day 81, with no clinically significant stricture during her remaining lifetime. At every endoscopic intervention, immediate hemostasis was visually confirmed before withdrawal of the endoscope (no active oozing or pulsatile bleeding from the treated site, and a stable clot at the treatment field); two interval re-bleeding episodes (days 54 and 71) each prompted repeat intervention with the methods described above. No further tube-related mucosal trauma was observed at subsequent surveillance OGDS.

During the healing phase her systemic sepsis escalated. On day 74 (9 February) the peritonitis evolved to an *Enterococcus* species, and on day 75 (10 February) she developed a catheter-related candidaemia (yeast isolated from the right internal jugular central-venous-catheter blood culture, with a negative concurrent peripheral culture). The central venous catheter was removed on day 78 (13 February) and antifungal therapy was given (intravenous anidulafungin 100 mg once daily, subsequently de-escalated to intravenous fluconazole). Despite these measures, source control of the intra-abdominal infection was not achieved. After multidisciplinary discussion with her family, a decision was made not to escalate further and dialysis was withdrawn (day 81); she died on day 91 (26 February 2026). The certified immediate cause of death was septic shock secondary to a refractory intra-abdominal infection and catheter-related candidaemia. Although the oesophageal mucosa was endoscopically healing by day 81, an indirect contribution from the prolonged and complicated oesophageal course (extensive necrotic injury, repeated endoscopic instrumentation, prolonged trans-oesophageal feeding-tube placement, and a transient suspected cavitation) cannot be entirely excluded.

## 3 Discussion

### 3.1 Spectrum of presentation and pathogenesis across the three cases

Although AEN is classically taught as a disease of older men with cardiovascular disease, contemporary systematic reviews emphasize its heterogeneity (6, 15, 18). The 2025 Kupferman pooled analysis confirmed male predominance and a median age in the seventh decade, but also highlighted that survival is influenced by individual patient factors more than by the oesophageal injury itself (6). Schizas et al. earlier synthesized 114 patients (83 men, 31 women) from the 1990–2018 literature, with 75.4% managed conservatively and in-hospital mortality of approximately 32% (15). Our three cases illustrate this breadth: Case 1 is the classical phenotype; Case 2 is the increasingly recognized variant in younger immunosuppressed hosts (11–13, 18); and Case 3 represents an emerging phenotype: severe recurrent-bleeding AEN in a long-term peritoneal-dialysis recipient with sepsis-driven critical illness.

Although the three cases share the unifying two-hit mechanism, the relative contributions of ischemia, anemia, and topical injury differ. In Case 1, the dominant pathogenic substrate was longstanding macrovascular and microvascular disease (type 2 diabetes and stage 3b chronic kidney disease) compounded by acute vomiting-induced gastric content reflux; hemodynamic instability was absent and the resulting injury was confined to the distal oesophagus. Cases 2 and 3 represent secondary hypoxic-ischemic injury in the context of systemic illness. In Case 2, several insults combined: profound hypoalbuminaemia (17 g/L), normocytic anemia, and vasopressor-supported septic shock reduced mucosal perfusion, while ciclosporin-induced microvascular vasoconstriction and the impaired mucosal repair associated with high-dose corticosteroid therapy further compromised mucosal defense and healing (12, 13). Case 3 illustrates the cumulative substrate of long-term dialysis: chronic anemia and uraemic mucosal dysfunction, repeated peritonitis-associated septic shock requiring intermittent vasopressor support, recurrent multidrug-resistant infections, and impaired mucosal repair (3, 14). Notably, although she had been a peritoneal-dialysis patient for 6 years, her Tenckhoff catheter had been removed and she had been converted to hemodialysis weeks before the oesophageal injury developed; the relevant predisposition was therefore the cumulative burden of dialysis-associated critical illness rather than the dialysis modality in use at the time. All three cases support the contention that anemia, hypoperfusion, and impaired mucosal defense act synergistically to precipitate AEN. AEN in patients on maintenance dialysis has been reported only rarely (14); our case adds to this limited literature and is, to our knowledge, among the first such reports from Southeast Asia.

### 3.2 Immunosuppression and renal failure as recognized substrates

Cases 2 and 3 emphasize the role of immunological compromise. Pharmacological immunosuppression has been implicated in solid-organ transplant recipients on calcineurin inhibitors and high-dose corticosteroids (12, 13), with mechanisms including calcineurin-inhibitor–induced microvascular vasoconstriction, steroid-mediated impairment of mucosal repair, and predisposition to opportunistic superinfection. Long-term dialysis confers a comparable but mechanistically different vulnerability through uraemic mucosal dysfunction, recurrent septic episodes, and chronic anemia (3, 14, 15). Clinicians caring for chronically immunosuppressed or dialysis-dependent patients with new-onset UGIB should include AEN in the differential diagnosis even when the demographic profile is atypical.

### 3.3 Endoscopic spectrum, differential diagnosis and superinfection workup

Diagnosis is established endoscopically by circumferential blackish mucosal discoloration that terminates abruptly at the gastro-oesophageal junction (2, 3, 9). The Gurvits staging system recognizes a progression from normal mucosa (Stage 0), through acute black necrosis (Stage 1), into a healing phase with pseudomembranes (Stage 2), to mucosal restoration (Stage 3), and finally to late stricture (Stage 4) (2). Case 1 demonstrated Stage 1 at presentation and Stage 3 by 6 weeks. Case 2 showed extensive Stage 1 at presentation, Stage 2 at day 17, and Stage 3 by day 76. Case 3 traced the same trajectory over a 32-day endoscopic course (Table 1), highlighting that the staging system applies even when the dominant phenotype is hemorrhagic and ulcerative rather than the prototypical diffusely black mucosa, and that severity correlates with the number of endoscopic interventions and time to healing rather than with case fatality.

Differential diagnoses for blackish or sloughy oesophageal mucosa include caustic ingestion, infectious oesophagitis (*Candida*, herpes simplex virus, and cytomegalovirus), oesophageal melanosis, primary or metastatic melanoma, pseudomelanosis from haemosiderin deposition, and drug- or radiation-induced injury (3, 9). Infectious oesophagitis is particularly important in immunosuppressed and critically ill patients. In Cases 2 and 3, mucosal biopsy was deferred during the acute phase because the perforation risk in friable necrotic mucosa was considered prohibitive (3, 9). However, surrogate evidence against active oesophageal superinfection was sought and documented in both: serial routine and fungal blood cultures, peripheral and central catheter-tip cultures, peritoneal-dialysate cultures (Case 3), oral and oropharyngeal *Candida* cultures, and cross-sectional imaging of the chest and mediastinum. Empirical antifungal therapy with fluconazole was continued in Case 3 throughout her admission. We acknowledge that a portal-of-entry contribution from the necrotic oesophageal segment to her later candidaemia cannot be entirely excluded (see Section 4.4).

### 3.4 Multimodal endoscopic hemostasis and prognosis

Treatment of AEN is principally medical: correction of the underlying systemic insult, bowel rest, high-dose parenteral proton pump inhibitor therapy, secure enteral access, and conservative endoscopic intervention (3, 6, 9, 19). The three cases here illustrate a hierarchy of management complexity. In Case 1, a focal bleeding point was readily controlled by submucosal adrenaline. In Case 2, diffusely friable necrotic mucosa precluded endoscopic hemostasis; an endoscopically placed nasojejunal tube secured distal enteral access. Case 3 represents the most complex endoscopic strategy in our series: recurrent hemorrhage required multimodal endoscopic hemostasis combining endoscopic clipping, submucosal adrenaline, topical hemostatic powder spray (Hemoblock® and Hemospray®), systemic tranexamic acid, and aggressive correction of coagulopathy. The cavitation observed on day 71 raised concern for an oesophago-respiratory or oesophago-pleural fistula; the negative under-water submersion test (no air bubbling) and subsequent uneventful healing argued against an established free communication. Fistulae remain an important rare complication and should prompt cross-sectional imaging and multidisciplinary input (9, 15, 19, 20). Surgical intervention to the oesophagus is reserved for perforation, contained mediastinal collection, or refractory bleeding (9, 15, 19, 20); none of our three patients required this.

The 2025 Kupferman systematic review identified advancing age and cardiovascular burden as predictors of worse outcome, with overall survival of 60.9% in the pooled cohort (6). These data, together with earlier reports in which the patient died from sepsis despite no further hematemesis (21), converge on a single contemporary teaching point: the principal determinant of outcome in AEN is the underlying systemic illness, not the oesophageal injury itself. Our series illustrates this strikingly. Case 1 had a favorable systemic profile and recovered uneventfully. Case 2 survived a critical-care course that was at least as dangerous as her AEN, and ultimately did well. Case 3 had the most extensive oesophageal disease of the three, the longest endoscopic course, and the most intensive endoscopic intervention; her oesophageal mucosa had endoscopically healed by day 81, and the certified cause of death 10 days later was septic shock from catheter-related fungaemia and intra-abdominal infection. However, given the prolonged complicated course, repeated endoscopic instrumentation, prolonged trans-oesophageal feeding-tube placement, and the transient suspected cavitation observed in the recovery phase, an indirect contribution from the necrotic oesophageal segment to the systemic septic course cannot be entirely excluded. Microbiological attribution of the fungaemia to a catheter-related source was supported by concordant central-line catheter-tip culture and matched blood-culture isolation; nevertheless, a contribution from the previously injured oesophagus, whether as a portal of microbial translocation or as a site of secondary colonization, remains an alternative or additive explanation that the available data cannot definitively exclude.

### 3.5 Strengths, limitations, and surveillance

Strengths of this series include the contemporaneous prospective endoscopic documentation of three contrasting AEN phenotypes managed in a single Southeast Asian center, a region under-represented in published AEN cohorts (6, 15), with complete sequential surveillance demonstrating mucosal recovery in every case. Limitations include the absence of histopathological confirmation in all three cases (the perforation risk of biopsy in friable necrotic mucosa was considered prohibitive) and the inherent constraints of a three-patient series. The most clinically important delayed complication is stricture (10–25% of survivors) (9, 15, 22); perforation occurs in approximately 7% and mediastinitis in approximately 6% (9, 15). All three of our patients demonstrated complete endoscopic mucosal restoration without stricture; in Case 3, a transient suspected cavitation did not progress to a clinical fistula.

### 3.6 Practical management considerations

Several practical lessons emerge from these three cases (the case-specific data are summarized in Table 2). First, bowel rest is appropriate during the acute hemorrhagic phase, but prolonged unnecessary fasting should be avoided; oral or enteral intake can be reintroduced once active bleeding has settled and surveillance endoscopy confirms healing, as in Cases 1 and 2. Second, where enteral access is needed in severe disease, post-pyloric nasojejunal feeding is preferable to nasogastric feeding because it minimizes the volume and acidity of gastro-oesophageal reflux on the injured mucosa, and is preferable to total parenteral nutrition because enteral feeding preserves gut-barrier function in critically ill septic patients and avoids the additional central-line burden; in our experience, endoscopic placement under direct vision is safe and was not associated with tube-related mucosal trauma at subsequent surveillance. Third, high-dose proton pump inhibitor therapy, given parenterally during the acute phase and continued orally during healing, is a consistent component of management, with the duration individualized to the severity and rate of mucosal recovery. Finally, surveillance endoscopy is valuable both to confirm healing and to detect the most important delayed complication, stricture; an initial surveillance procedure at 4–8 weeks is reasonable in uncomplicated cases, with additional procedures in severe or complicated disease, as required in Case 3 (3, 9, 23–25).

**Table 2:** Comparison of clinical, endoscopic, and management features of the three cases.

**Feature**	**Case 1**	**Case 2**	**Case 3**
Age, sex	77, male	52, female	49, female
Background comorbidity	T2DM; CKD 3b	Relapsed FSGS on prednisolone 45 mg/d + ciclosporin 50 mg BD; CKD; recent herpes zoster	ESRF on CAPD 6 y; recurrent *A. baumannii* peritonitis; Tenckhoff removed and converted to HD during admission
Anticoagulation/antiplatelet	None	None	None
Acute trigger	Vomiting; haemodynamically stable	MSSA bacteraemia + HAP; intubated; vasopressor-dependent; haematemesis prompting OGDS	Relapsing *A. baumannii* peritonitis at admission; later CP-CRE Klebsiella + intra-abdominal collection; Sepsis-3; RICU; UGIB on day 49
Endoscopic findings	Circumferential blackish mucosa GEJ to mid-oesophagus; longitudinal break with exposed muscle and oozing	Massive intraluminal clots; extensive submucosal tear with intramural haematoma; multifocal necrosis	Large 15-cm adherent clot; sloughy necrotic mucosa 22–28 cm; F3 longitudinal ulcers; transient suspected cavitation
Number of OGDS	2 (index + 6-week surveillance)	3 (days 0, 17, 76)	8 (days 49–81; see Table 1)
Endoscopic intervention	Submucosal adrenaline 1:10,000	None to necrotic segment; endoscopic NGJ for distal feeding	4 endoclips; submucosal adrenaline; Hemoblock®; Hemospray®; IV tranexamic acid; coagulopathy correction; endoscopic NGJ via enteroscopy
Acid suppression/mucosal protection	IV esomeprazole 72 h → oral 40 mg BD × 8 wk.; sucralfate × 4 wk	IV esomeprazole 8 mg/h × 48 h → 40 mg BD × 12 wk	IV esomeprazole 8 mg/h × 72 h during each bleed → 40 mg BD between
Nutritional route	Bowel rest 72 h → soft diet day 4	Continuous NGJ days 1–60; PN supplementation in ICU; oral intake from day 60	NBM 22 days → NGJ to death; PN bridge during re-bleeds
Time to mucosal healing	6 weeks	~10 weeks (76 days)	32 days from first OGDS
Length of stay	8 days	92 days	91 days
Final outcome	Full recovery	Full oesophageal recovery; ongoing FSGS follow-up	Mucosal healing achieved; died day 91 of septic shock (refractory intra-abdominal infection + catheter-related candidaemia); indirect oesophageal contribution cannot be excluded

BD, twice daily; CAPD, continuous ambulatory peritoneal dialysis; CKD, chronic kidney disease; CP-CRE, carbapenemase-producing Enterobacteriaceae; ESRF, end-stage renal failure; FSGS, focal segmental glomerulosclerosis; GEJ, gastro-oesophageal junction; HAP, hospital-acquired pneumonia; IV, intravenous; MSSA, methicillin-sensitive *Staphylococcus aureus*; NBM, nil by mouth; NGJ, nasojejunal; OGDS, oesophagogastroduodenoscopy; PN, parenteral nutrition; RICU, renal intensive care unit; T2DM, type 2 diabetes mellitus.

## 4 Patient perspective

Case 1: “I was shocked when the doctors told me my oesophagus was black and bleeding. I am thankful that, with rest and medication, everything healed completely.”

Case 2: “I remember being told I had bleeding in my food pipe at a time when I was already very unwell with infection. I am grateful that the doctors chose to treat me with rest and feeding through a tube rather than operate, and that my oesophagus and my kidneys eventually got better.”

Case 3 (relayed by the patient’s next of kin): “My sister fought through so many endoscopies and bleeding episodes; we are comforted that, even though we lost her to infection in the end, her oesophagus had finally healed. Sharing her story may help other patients on long-term dialysis.”

## 5 Conclusion

Acute oesophageal necrosis is a rare but potentially fatal cause of upper gastrointestinal bleeding that should be considered in any acutely unwell patient with hematemesis or coffee-ground emesis, regardless of demographic profile. Our three cases illustrate the clinical breadth of the entity, from a relatively well elderly comorbid man, to a younger immunosuppressed woman with sepsis-driven critical illness, to a long-term peritoneal-dialysis recipient with recurrent peritonitis whose disease required eight endoscopic procedures and multimodal hemostasis before complete mucosal healing. The first two patients recovered. The third died on day 91 of admission; although her oesophageal mucosa was endoscopically healed and the certified cause of death was septic shock from catheter-related fungaemia and intra-abdominal infection, an indirect contribution from the prolonged and complicated oesophageal course cannot be excluded. Successful management depends on aggressive correction of the underlying systemic insult, supportive measures including bowel rest, acid suppression, and secure enteral access, and judicious endoscopic intervention when bleeding is amenable to local hemostasis. Surveillance endoscopy should be considered, particularly in severe or complicated cases, to confirm healing and detect early stricture. Above all, our series reinforces the contemporary teaching that prognosis in AEN is determined principally by the underlying systemic illness rather than by the oesophageal injury itself.

## Data Availability

The original contributions presented in the study are included in the article/supplementary material, further inquiries can be directed to the corresponding author/s.
